# Case of Hydrophobia

**Published:** 1821-04

**Authors:** William Mines

**Affiliations:** Surgeon.


*Case of Hydrophobia.* By William Mines, Surgeon.

ROBERT MERRY, footman to Robert Sheriffe, Esq. of Diss, applied to me for medical aid, December 1st, 1820, eight o'clock, A.M. complaining of having had a very restless night: he could get no sleep, from unpleasant symptoms about his throat, with extreme difficulty of swallowing even his saliva; and at times his breathing was much oppressed. His pulse was under

70, soft and regular, without fever and, apparently, any inflammatory action. A draught, with camphor julep, &c. was poured out for him, which he appeared very anxious to take; but struggled much from every attempt, declaring he could not swallow. However, with great difficulty a tea-spoonful or two was got down, which brought on considerable spasm about the throat, so that all subsequent efforts to get down food or liquid of any kind were fruitless, as every attempt brought on most violent startings; and even the name or sight of water was horrid to him; and, when poured from one vessel to another, it instantly threw him into strong convulsions.

Full forty ounces of blood were taken from the arm without producing syncope, or even any particular change or diminution of symptoms, and the pulse varied very little from the loss of blood. During the bleeding, and for a short time after, he several times called out he thought he was the better for it; but, as the symptoms did not abate, and the blood had not the slightest inflammatory appearance, it was not thought advisable to repeat the bleeding.

*Five o'clock, p. m.*—Dr. Shorting saw this patient; and, after witnessing the symptoms as before described, believed it to be a true case of hydrophobia, and, although no reliance could be placed in any thing, was anxious that mercurial frictions should be used largely, and considerable doses of laudanum given by injections; neither of which produced any sensible effect.

During this disease the patient was at intervals perfectly calm and rational, answering all questions, till nearly the last, when his convulsions became so strong that it was thought necessary to put on the strait-waistcoat for safety; to which he made no resistance. There had been no slaver about the mouth, till nearly the last, when, half an hour before his dissolution, he vomited up several spoonfuls of froth; and a few minutes after died without a groan or a struggle, (six o'clock, A.M. Dec. 2d,) labouring under this dreadful disease in its active form only twenty-two hours.

This poison must have been received about three weeks prior to the attack, at which time the servant had waited upon a terrier that died with the disease, having been bitten by a mad dog passing through this town the latter end of July last; which terrier had likewise worried and slavered a spaniel over, that the servant assisted in wiping and cleaning; but from neither of the animals did he receive any bite, nor had he apparently any abrasion or scratch of the skin.

*Diss; January* 1821.

P.S.—I regret I am not able to add the appearances on dissection: permission to open the body having been denied by the patient's relatives.

